# Tetrandrine, a Chinese plant-derived alkaloid, is a potential candidate for cancer chemotherapy

**DOI:** 10.18632/oncotarget.8315

**Published:** 2016-03-24

**Authors:** Ting Liu, Xin Liu, Wenhua Li

**Affiliations:** ^1^ College of Life Sciences, Wuhan University, Wuhan, P. R. China; ^2^ Ministry of Education Laboratory of Combinatorial Biosynthesis and Drug Discovery, College of pharmacy, Wuhan University, Wuhan, P. R. China

**Keywords:** tetrandrine, natural product, cancer, chemotherapy

## Abstract

Cancer is a disease caused by the abnormal proliferation and differentiation of cells governed by tumorigenic factors. Chemotherapy is one of the major cancer treatment strategies, and it functions by targeting the physiological capabilities of cancer cells, including sustained proliferation and angiogenesis, the evasion of programmed cell death, tissue invasion and metastasis. Remarkably, natural products have garnered increased attention in the chemotherapy drug discovery field because they are biologically friendly and have high therapeutic effects. Tetrandrine, isolated from the root of *Stephania tetrandra* S Moore, is a traditional Chinese clinical agent for silicosis, autoimmune disorders, inflammatory pulmonary diseases, cardiovascular diseases and hypertension. Recently, the novel anti-tumor effects of tetrandrine have been widely investigated. More impressive is that tetrandrine affects multiple biological activities of cancer cells, including the inhibition of proliferation, angiogenesis, migration, and invasion; the induction of apoptosis and autophagy; the reversal of multidrug resistance (MDR); and the enhancement of radiation sensitization. This review focuses on introducing the latest information about the anti-tumor effects of tetrandrine on various cancers and its underlying mechanism. Moreover, we discuss the nanoparticle delivery system being developed for tetrandrine and the anti-tumor effects of other bisbenzylisoquinoline alkaloid derivatives on cancer cells. All current evidence demonstrates that tetrandrine is a promising candidate as a cancer chemotherapeutic.

## INTRODUCTION

Cancer is a disease caused by the abnormal proliferation and differentiation of cells governed by tumorigenic factors. There are more than 100 diverse types and subtypes of cancer that can be found within specific organs. Most human tumors are characterized by six physiological capabilities that occur during tumor development, including self-sufficiency in growth signals, insensitivity to growth-inhibitory signals, evasion of programmed cell death, limitless replicative potential, sustained angiogenesis, and tissue invasion and metastasis [1]. Cancer is the second most common cause of death worldwide.

Chemotherapy is a major strategy for cancer treatment. Unlike surgery and radiotherapy, which target a particular body part to remove or kill cancer cells, chemotherapy works with lower negative impact on the entire body to inhibit tumor cells effectively, especially to prevent cancer cells from spreading to other parts of the body through metastasis. There are currently approximately 90 types of chemotherapy drugs widely used in cancer treatment. These chemotherapeutic agents have been classified as alkylating agents, antimetabolites, platinum compounds, antitumor antibiotics and natural products. Historically, natural products, which are fairly pure compounds extracted from plants, have been major sources of therapeutic formulations for disease. Because natural products are considered to have co-evolved with their target sites in biological systems, the application of natural products for the control of cancer is viewed as better and often more biologically friendly in the field of drug discovery than are non-natural products [2]. Natural products have garnered increasing attention in cancer chemotherapy, including harringtonine, camptothecin and Ptx, which are all traditional natural products for cancer treatment. The famous 2015 Nobel Prize in Physiology or Medicine was awarded for the discovery of Qinghaosu (artemisinin), which is extracted from the herb *Artemisia annua*, is an antimalarial drug from China and is undergoing early research and testing for the treatment of cancer.

**Figure 1:**
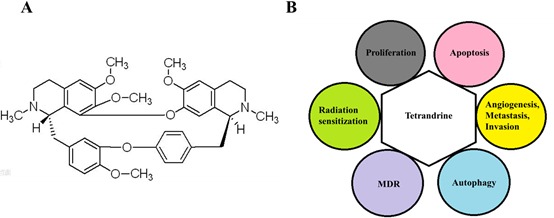
**A.** Structure of tetrandrine. **B.** Effects of tetrandrine on cancer cells. Tetrandrine has numerous effects on cancer cells, including the inhibition of proliferation, angiogenesis, migration, and invasion, the induction of apoptosis and autophagy, the reversal of MDR, and the enhancement of radiation sensitization.

Tetrandrine is one type of natural product originally extracted from Chinese herbs. Tetrandrine [(1b)-6,6′,7,12-tetramethoxy-2,2′-dimethyl-berbaman] (Figure 1A), which belongs to the bisbenzylisoquinoline alkaloid family and was isolated from the root of *Stephania tetrandra* S Moore, possesses multiple pharmacologically relevant classes. As a clinical drug in China, tetrandrine has been used for decades to treat patients with silicosis, autoimmune disorders, inflammatory pulmonary diseases, cardiovascular diseases and hypertension. Several early studies found that tetrandrine has pharmacological potential in cancer therapy. The most beneficial effects of tetrandrine on tumor cells is the inhibition of proliferation and the induction of apoptosis, not only on cancer cell lines, such as human leukemic U937 [3], human hepatoma HepG2 [4], human lung carcinoma A549 [5], and human colon cancer HCT-116 [6] cells but also on primary cancer cells isolated from ascites and pleural fluids, such as A-Ga31, A-Li40, P-Lu18 and A-Co20 cells, which were isolated from patients with gastric, liver, lung and colon cancers, respectively [7]. Other bioactivities of tetrandrine include the reversal of multidrug resistance (MDR) [8–11], the sensitization of tumor cells to radiation radiosensitization, and the inhibition of angiogenesis and metastasis. However, the mechanisms of these effects were not clearly addressed in publications at that time. Recently, numerous studies have been successfully performed to reveal the anti-tumor bioactivity of tetrandrine. In particular, many studies specifically investigated the mechanisms underlying tetrandrine treatment for various cancer cells (Table 1 and Table 2). The autophagy induction capacity of tetrandrine and a method of delivering tetrandrine to cancer cells effectively have recently been developed. Additionally, to improve the anti-tumor efficiency of tetrandrine, increasing evidence has suggested developing nanoscale delivery systems for the delivery of tetrandrine into cancer cells. Notably, other bisbenzylisoquinoline alkaloid derivatives, such as fangchinoline, also have effects on cancer cells.

**Table 1:** Effects of tetrandrine treatment alone on cancer cells

Cancer types	Name of cell line (concentration)	Therapeutic effects	Mechanism	References
Oral cancer	SAS (25 μM), HSC-3 (20 μM)	Autophagy, Apoptosis	BeclineI/LC3-I/II, PARP, Caspases	[35, 36]
Prostate cancer	PC3 (15 μM), DU145(15 μM)	Apoptosis, Autophagy, Metastasis, Invasion, Proliferation	Caspases, ROS/JNK1/2, PI3K-Akt	[37, 38, 126]
*Lung carcinoma	A549 (30 μM)	Apoptosis, Proliferation, Autophagy	P21, Akt, ERK, ROS	[43, 44, 69]
Gastric Cancer	BGC-823 (8 μg/ml)	Apoptosis	Mitochondria/ Caspases	[47]
*Breast cancer	4T1(1 μM), SUM-149(1 μM), SUM-159(2 μM), MCF-7(12 μM), MDA-MB-231(12 μM)	Metastasis, Angiogenesis, Mammosphere, Proliferation, Autophagy	p-ERK, NF-κB, Metastatic and angiogenic related proteins, ROS	[29, 61, 69, 125, 128]
Renal cell carcinoma	786-O(15 μM), 769-P(15 μM) ACHN (15 μM)	Apoptosis, Cell cycle arrest	Caspases, p21 and p27	[27]
* Hepatic carcinoma	HepG2(5 μM), Hep3B(10 μM), PLC/PRF/5(10 μM), Huh-7(20 μM)	Apoptosis, Autophagy, Cell cycle arrest,	Mitochondria/ Caspases, ROS	[24–26, 45, 46, 70]
Colon cancer	CT-26(10 μM), LoVo(15 μM), HT29(15 μM), HCT116(5 μM)	Apoptosis, Invasion, Metastasis, Cell cycle arrest	p38 MAPK, IGFBP-5, Wnt/β-catenin, E2F1, p53/p21, PI3K/AKT/GSK3β	[23, 39–42, 59]
Bladder cancer	5637(20 μM), T24(20 μM)	Apoptosis	Mitochondria/ Caspases	[48]
*Glioma	RT-2, U87(20 μM)	Apoptosis, Autophagy Angiogenesis, Proliferation, Metastasis, Invasion	Caspases, eIF-2α, VEGF, ADAM17/ EGFR-PI3K/AKT, ROS	[49, 58, 60, 69]
Hemangioendothelioma	EOMA(50 μM)	Apoptosis, Proliferation	ROS/Akt	[28]
Nasopharyngeal carcinoma	CNE (30 μM)	Apoptosis		[105]
*Cervical carcinoma	HeLa(5 μM)	Autophagy	ROS	[69]
* Acute melocytic leukemia	NB4 (2 μM)	Autophagy, Differentiation, Proliferation	ROS/Notch1	[71]

* Studies involving our laboratory

Metabolism, pharmacokinetic and toxicology studies play important role in the discovery and development of drugs. Pharmacokinetic and toxicokinetic properties of tetrandrine are essential to its further research for clinical use. The pharmacokinetical study of tetrandrine with a simple HPLC method in rabbits showed that the concentration-time data of tetrandrine fit the classical two-compartment model, no matter the drug was administered intravenously or orally to rabbits and tetrandrine displays a limited absorption in intestinal tract [12]. With a liquid chromatography-tandem mass spectrometric method for the determination of tetrandrine in rat plasma after a single oral administration (50 mg/kg), tetrandrine was found no sexual difference in pharmacokinetics of tetrandrine in rats [13]. Besides its pharmacological effects, some data from the animal studies indicated the potential accumulation and injury to human livers from chronic administration of tetrandrine. Tetrandrine was reported for its liver toxicity when administered in high dose for a relatively long period in dogs [14] and daily administration of tetrandrine (57 mg/kg by oral gavage) for 8 d can induce obvious liver injury in rats [15]. It is relatively non-toxic to humans, even at the administration of 180 mg, intramuscularly three times daily [16, 17]. The researchers for Ebola virus disease take tetrandrine as the best candidate for animal testing because of low cytotoxicity [18].

Accumulated evidence from basic research has proven that tetrandrine has significant anti-tumor effects on various cancer cells *in vitro* and *in vivo*. In this review, we discuss the effects of tetrandrine on various cancer therapies as well as its underlying mechanism. The inhibition of proliferation, angiogenesis, metastasis, and invasion; the induction of autophagy and apoptosis, and even differentiation; the reversal of MDR; and the enhancement of radiation sensitization in cancer cells all indicate that tetrandrine is a potential antineoplastic drug (Figure 1B). In addition, we discuss the development of a nanoparticle delivery system for tetrandrine and the anti-tumor effects of other bisbenzylisoquinoline alkaloid derivatives on cancer cells. All current evidence demonstrates that treatment with either tetrandrine alone (Table 1) or in combination with other chemotherapeutic agents (Table 2) is a potential chemotherapy strategy for cancer.

**Table 2:** Effects of tetrandrine treatment in combination with other agents on cancer cells

Name of agent	Cancer cell line	Therapeutic effects	References
5-FU	HCT116	β-catenin – Migration and Invasion	[39]
Imatinib	K562, primary leukemia cells	G1 arrest, Depletion of p210 (Bcr-Abl) mRNA and β-catenin protein	[92]
Endostar	LoVo, human colon cancer	Apoptosis, Cell cycle arrest, Angiogenesis, Metastasis	[102]
Docetaxel	KBv200	Inhibition of P-gp, Apoptosis	[85]
Vincristine	KBv200	Binding to P-gp- reversed resistance	[86]
Doxorubicin	Caco-2, CEM/ADR5000, MOLT-4/DNR, K562	Reducing P-gp expression, inhibition of mmdr1, mRNA/P-gp and NF-kappaB	[87, 88, 91]
Daunorubicin	MOLT-4/DNR, K562/A02	Down-regulating GCS- Apoptosis	[88, 90]
Vinblastine	MOLT-4/DNR	Inhibition of P-gp	[88]
Daunorubicin, Etoposide and Cytarabine	acute myeloid leukemia	More tolerated in clinical trial for patients	[89]
Cisplatin	YES-2/DDP, ovarian cancer,	Wnt/cadherin-apoptosis, Down-regulation of MRP1	[94, 95]
* Sorafenib	BEL7402, FHCC98, HepG2, HCT116, RKO, DLD1	ROS/Akt, Apoptosis	[52]
Arsenic trioxide	HepG2, A549	Apoptosis, necrosis and cell cycle arrest	[100]
* Chloroquine	Huh7, FHCC98, U87, U251, Calu-1, A549, HeLa	ROS/p21CIP1/WAF1- Apoptosis	[75]
PTX and PTX loaded nanoparticles	KBv200, gastric cancer	ROS, Apoptosis	[85]
Lactoferrin-conjugated polymer, some holding doxorubicin (Lf-PO-Dox)	C6 glioma cells	Inhibition of tumor growth	[93]

* Studies involving our laboratory

## INHIBITING CANCER CELL PROLIFERATION

In tumor progression, unrestrained cell proliferation resulting from disordered cell division is frequently accompanied by cell cycle dysregulation [19]. The cell cycle control that is associated with cancer occurs through cyclin-dependent kinases (CDK)/Cyclin kinase hyperactivity at different stages of the cell cycle. In cancer, mutations have been observed in genes encoding CDK, cyclins, CDK-activating enzymes, CKI, CDK substrates, and checkpoint proteins [20]. Because CDKs are critical regulators of cell cycle progression, cyclin-dependent kinases represent attractive pharmacological targets for the development of anticancer drugs, and targeting CDKs has been pursued as a strategy for therapeutic intervention [21]. The inhibition of these CDKs primarily reduces the accumulation of transcripts with short half-lives, including those encoding anti-apoptotic family members and cell cycle regulators, as well as p53 and nuclear factor-kappa B-responsive gene targets [22].

Early reports demonstrated that tetrandrine-induced G1 arrest in human colon carcinoma cells is mediated by the inhibition of cyclin-dependent kinase, the down-regulation of E2F1 and the up-regulation of p53/p21(Cip1) [6]. Tetrandrine also induced G1 arrest in human colon carcinoma HT-29 cells through the PI3K/AKT/GSK3beta pathway *via* down-regulation of cyclin D1 as well as up-regulation of p27(kip1) [23]. The Ng group evaluated the effects of tetrandrine on three different hepatoma cell lines, HepG2, PLC/PRF/5 and Hep 3B, and found that the effects of tetrandrine on cell cycle progression varied with hepatoma cell type. The HepG2 and PLC/PRF/5 cells treated with tetrandrine arrested at the G2/M phase in a dose-dependent manner but not the Hep 3B cells [24]. Moreover, Huh-7 hepatocellular carcinoma cells treated with tetrandrine suppress cell cycle progression at the G2/M phase [25]. In contrast, tetrandrine showed marked induction of G0/G1 cycle arrest in HepG2 and PLC/PRF/5 cells in a study by Wei et al. [26]. In addition, Chen et al. clarified the underlying mechanisms of the anticancer effect of tetrandrine in human renal cell carcinoma (RCC) 786-O, 769-P and ACHN cells. Their study showed that tetrandrine was capable of inducing G1 cell cycle arrest in RCC cells regulated by cell cycle protein p21 WAF1/CIP1 and p27(kip1) [27]. Recently, in mouse endothelial cells (EOMA cells), tetrandrine exhibited significant proliferation inhibition and G1/S arrest *via* down-regulated expression of cyclin D, cyclin E and CDKs. Furthermore, intracellular accumulation of reactive oxygen species (ROS) and the decline of phospho-Akt protein levels play an important role in tetrandrine-induced cell cycle arrest [28]. Specifically, tetrandrine inhibits the proliferation of SUM-149 and SUM-159 breast cancer initiation cells (TICs). Tetrandrine also inhibited mammosphere formation of breast cancer TICs *in vitro* and had similar effects on mammosphere formation in cells isolated from fresh patient samples [29]. Taken together, all evidence provided suggests that inhibiting proliferation is one of the effects of tetrandrine in cancer cells.

## INDUCING APOPTOSIS OF CANCER CELLS

The characteristics of cancer cells stem from not only the amplification of positive growth signals, the mutation of checkpoint and surveillance genes but also the deregulation of cell death. These characteristics promote inducing cancer cells to death as a critical strategy for cancer therapy.

Apoptosis, also called type I programmed cell death, is a form of programmed cell death that occurs after receiving specific stimuli, which are characterized by morphologic changes that include chromatin condensation, nuclear fragmentation and the reduction of cell volume, as well as biochemical changes including caspase activation, the breakdown of DNA, and protein and membrane surface modifications [30, 31]. Apoptosis may be triggered by two complementary extrinsic and intrinsic pathways: one involves the binding of death ligands to death receptors and the other (intrinsic) initiates the “mitochondrial” pathway [32]. Many tumor cells develop mechanisms to evade apoptosis, such as the expression of anti-apoptotic proteins or the down-regulation or mutation of pro-apoptotic cell death components [33]. Thus, targeting the apoptosis pathway is one of the important chemotherapeutic strategies for malignant tumors, and this pathway is the major type of cell death induced by most of the frontline chemotherapeutic agents [34]. Numerous studies have reported that tetrandrine induced apoptosis effects in many human cancer cells.

Furthermore, tetrandrine significantly decreased the viability of human oral cancer SAS [35] and HSC-3 [36] cells in a concentration- and time-dependent manner. By inducing chromatin condensation, inter-nucleosomal DNA fragmentation, activation of caspase-3, caspase-8, and caspase-9, and cleavage of poly (ADP ribose) polymerase (PARP), tetrandrine induces apoptosis of human oral cancer cells *via* the caspase-dependent pathway in these studies.

Tetrandrine also exerts an apoptosis effect on human prostate cancer PC3 and DU145 cells that is mediated by both the intrinsic and extrinsic pathways. The specific molecular mechanisms have been elucidated. Tetrandrine-mediated ROS generation is caused by the activation of c-Jun NH2-terminal kinase (JNK1/2), which mediates proteasomal degradation of the c-FLIP L/S and Bcl2 proteins. Degradation of these proteins causes the prostate cancer cells to undergo Fas- and mitochondria-mediated apoptosis and the induction of ligand-independent Fas-mediated apoptosis by activating procaspase-8 and Bid cleavage in parallel [37]. Liu and colleagues also confirmed that tetrandrine induced apoptosis in prostate cancer PC3 and DU145 cells in a dose-dependent manner by activating the caspase cascade and inhibiting the phosphoinositide 3-kinase-Akt signal pathway [38].

By comparing tetrandrine with six chemotherapy drugs in eight cancer lines, He et al. found that tetrandrine exhibits anticancer activities comparable to those of camptothecin, vincristine, paclitaxel, and doxorubicin and that these activities were better than those of 5-fluorouracil (5-FU) and carboplatin. Tetrandrine-induced apoptosis may be at least in part mediated by catenin-targeting activity [39]. In the process of tetrandrine-induced apoptosis in colon cancer HT-29 cells, GSK3beta is activated *via* AKT inhibition, resulting in the activation of caspase 3 and subsequent cleavage of PARP [23]. Wu et al. determined that tetrandrine induced significant apoptosis of cultured and subcutaneous colon cancer CT-26 cells in a concentration- and time-dependent manner. This apoptosis effect may be associated with activation of the p38 MAPK signaling pathway, leading to slower tumor growth, longer animal survival time and higher survival rate [40]. Tetrandrine has also been reported to induce apoptosis *in vitro* and *in vivo*, accompanied by the formation of ROS and the activation of ROS-dependent c-JNK and caspase-3 in colon cancer LoVo cells [41]. Recently, one study revealed that tetrandrine-induced apoptosis in LoVo cells may be partly related to the down-regulation of IGFBP-5 expression, thus inactivating Wnt/β-catenin signaling transduction [42].

Liou et al. elucidated the molecular mechanism of apoptosis induction in lung cancer cell line A549 that involves the up-regulation of cyclin-dependent kinase inhibitor p21, which then mediates the activation of caspase-3 and subsequent down-regulation of cyclin D1 [43]. In addition, ERK phosphorylation was also down-regulated in tetrandrine-induced apoptosis in A549 human lung carcinoma cells in both time- and concentration-dependent manners [44].

Moreover, tetrandrine exhibits nuclear fragmentation and apoptotic features in HepG2, Huh-7 and PLC/PRF/5 hepatoma cells [24, 26]. Furthermore, tetrandrine-induced apoptosis in HepG2 and Huh-7 cells was caused by p53 up-regulation, Bcl-XL down-regulation, Bid and Bax cleavage, and the release of cytochrome c, which are all associated with caspase cascade activation [25, 45]. There is also *in vitro* and *in vivo* evidence of the apoptosis effects of tetrandrine on Huh7, HepG2 and BEL7402 human hepatocellular carcinoma cells through the regulation of Bcl-2 family proteins and the activation of caspase cascades by activating reactive oxygen species and repressing Akt activity [46].

By investigating the effects of tetrandrine on human gastric cancer BGC-823 cells, Qin R et al. reported tetrandrine-induced apoptosis of BGC-823 cells *in vitro* and *in vivo*. The up-regulation of Bax, Bak, and Bad and the down-regulation of Bcl-2 and Bcl-xl, which are related by the mitochondrial pathway, both contribute to tetrandrine-induced apoptosis in dose- and time-dependent manners. Moreover, tetrandrine effectively inhibited tumor growth *via* the induction of apoptosis in a nude mouse xenograft model [47]. Other studies revealed that tetrandrine was capable of triggering apoptosis in 5637 and T24 human bladder cancer cells and in 786-O, 769-P and ACHN human RCC cells *in vitro*, which was accompanied by the activation of a very strong and prominent caspase cascade and the mitochondrial pathway in a concentration-dependent manner [27, 48]. Additionally, tetrandrine effectively induced the apoptosis of glioma cells in concentration- and time-dependent manners [49] and induced the apoptosis of EOMA cells *in vitro* and *in vivo* [28].

Agents that selectively kill or sensitize tumor cells with no or low additional toxicity to normal cells would have significant value on clinical therapies. Interesting, tetrandrine causes no significant addition of toxicity to normal cells compared to tumor cells. 6-15μΜ tetrandrine induced no apparent apoptosis in MCF10A cells (a non-tumorigenic epithelial cell line) and normal prostate epithelial PWR-1E cells only showed less apoptosis (2.9 to 9.5%) at 30μΜ concentration of tetrandrine exposure [50, 51]. Also, immortalised non-malignant human mammary epithelial HBL100cells and normal human hepatic L02 cells were less sensitive to tetrandrine treatment [52]. In addition, low concentration (2 micrograms/ml) can protect normal human mononuclear cells *in vitro* against damage from a single high-dose of ionizing irradiation (10 Gy) [53].

## BLOCKING ANGIOGENESIS, MIGRATION AND INVASION OF CANCER CELLS

Tumor angiogenesis, the growth of new blood vessels for tumors, is considered an essential pathological feature of cancer as growing tumors need additional nutrients and oxygen. Tumor angiogenesis is also indispensable for enabling other aspects of tumor pathology such as metabolic dysregulation and tumor metastasis and invasion [54]. Tumor metastasis and invasion are major causes of mortality in cancer patients. Because of the complex interplay between tumors and stromal cells, including endothelial cells and associated mural cells, resistance against antiangiogenic therapies is gaining increasing awareness, especially for highly vascularized tumors such as HCC [55]. Most agents target the well-known vascular endothelial growth factor pathway, and some extracellular molecules target the inhibition of invasion [56, 57].

Because tetrandrine is a highly lipid-soluble, hydrophobic molecule with a low molecular weight, it may cross the blood brain barrier. Chen et al. investigated the effects of tetrandrine (150 mg/kg/day) on the inhibition of angiogenesis in subcutaneous RT-2 glioma cells by inhibiting the expression of VEGF in glioma cells. Tetrandrine also affected intracerebral tumors, showed cytotoxicity on the ECV304 human umbilical vein endothelial cells and suppressed *in vivo* angiogenesis [49, 58]. The anti-metastatic effect of tetrandrine was investigated in a pulmonary metastatic model of colorectal adenocarcinoma-bearing BALB/c mice after murine colorectal adenocarcinoma CT26 cells were injected into BALB/c mice *via* the tail vein to establish pulmonary metastases [59]. Additionally, in U87 glioma cells, one study explored the ability of tetrandrine to inhibit cell migration and invasion *in vitro* [60]. Gao and colleagues exhibited the anti-angiogenic and anti-metastatic activities of tetrandrine in a 4T1 tumor-bearing BALB/c mice model to be even better than those of doxorubicin. Moreover, tetrandrine significantly inhibited endothelial cell proliferation, adhesion, migration, invasion, and tube formation by targeting vascular endothelial growth factor, hypoxia-inducible factor-1, integrin 5, endothelial cell specific molecule-1, and intercellular adhesion molecule-1 *in vivo* [61]. As an inhibitor of tumor vascular growth, tetrandrine was verified to have antiangiogenic effects *in vivo* in a liver cancer nude mice xenograft model [28] and significantly weakened the migration and invasion capacity of DU145 and PC-3 human prostate cancer cells [38].

## INDUCING CANCER CELL AUTOPHAGY

Macroautophagy (referred to as autophagy here) is a dynamic process mediated by cellular components in the cytoplasm, including proteins and organelles engulfed by double-membrane autophagosomes that are subsequently delivered to the lysosome for degradation and recycling [62, 63]. Autophagy acts as a balancing mechanism between cell survival and cell death (autophagic cell death) [64]. In normal cells, the survival function is particularly important during development and under certain environmental stress conditions, and cellular death is critical during processes that involve extensive cellular remodeling [65]. Evidence has shown that autophagy is important in normal growth control and may be defective in cancer cells [66, 67]. In cancer cells, autophagy has a dual role, which as a critical pathway in tumor development and tumor therapy. Therapeutics targeting autophagy could be inhibitory to induce non-autophagic forms of cell death or stimulatory to incite autophagic cell death [68].

Tetrandrine has been proven to be a potent broad-spectrum autophagy agonist with effects on a variety of cell lines, including MCF-7, HeLa, PC3, U87, MDA-MB-231, and A549 cancer cells, and HFF and HEK 293 nontumorigenic cells, and it exhibits a much stronger activity in inducing autophagy than the autophagy activator rapamycin [69]. Additionally, tetrandrine has the capability to induce autophagy in human hepatocellular carcinoma Huh7, BEL7402, HepG2, and L02 cells *in vitro* and *in vivo*, and the autophagy-inducing activity is at least partially dependent on the accumulation of intracellular ROS and the repression of ATG7 [70]. In addition, tetrandrine-induced autophagy in human oral cancer HSC-3 cells and SAS cells occurs *via* Becline I/LC3-I/II dependent signaling pathways [35, 36]. The efficacy of tetrandrine on NB4 leukemia cells *in vivo* and *in vitro* revealed that tetrandrine-induced differentiation is accompanied by autophagy from the accumulation of ROS and by Notch1 signaling activation. Furthermore, autophagy and differentiation were also induced by tetrandrine in M5 type patient primary leukemia cells [71]. Different from an autophagy activator, Qiu et al. presented tetrandrine as a potent lysosomal inhibitor, blocking autophagic flux at the lysosomal degradation stage in tumor cells, predominantly by neutralizing lysosomal acidity and then decreasing the recycling of cellular fuels [72].

On the one hand, tetrandirne-induced cell death may act through autophagy (autophagic cell death) in cancer cells [35, 36, 73]. On the other hand, the tetrandirne-induced autophagy may play a protective function and weaken apoptosis in tumor cells which provide an efficient approach to synergize apoptosis by pharmacologically inhibiting autophagy [71, 74, 75].

## REVERSAL OF MULTIDRUG RESISTANCE

Multidrug resistance to anticancer drugs is one of the major obstacles to successful tumor chemotherapy. Of the potential mechanisms of MDR cancer cells, the most predominantly reported ones include the adenosine triphosphate (ATP)-binding cassette (ABC) transporter family [76], apoptosis induction [77], autophagy induction [78], cancer stem cell regulation [79], miRNA regulation [80], hypoxia induction [81], DNA damage and repair [82], and epigenetic regulation [83]. Currently, looking for novel compounds with anti-MDR activity is a promising approach to solving this cancer drug resistance problem; in combination with anticancer drugs, these compounds may increase the anticancer effect, which has led to increased intracellular drug retention and the recovery of cell sensitivity to chemotherapeutic drugs in combined treatment. Moreover, some naturally occurring compounds may be used as chemosensitizers in the treatment MDR cancers.

MDR gene product P-glycoprotein (P-gp), a well-known ABC transporter family member, is a drug efflux pump that mediates MDR by decreasing the intracellular concentration of cancer drugs; however, it can be inhibited by compounds with a variety of pharmacological effects to circumvent the MDR phenotype [84]. Thus, the development of effective transporter inhibitors could be valuable to cancer treatment. Tetrandrine is a good candidate for the development of new MDR-reversing agents, and its reversal mechanism is most likely due to the potent inhibition of P-gp. Co-administration of tetrandrine significantly reversed the sensitivity of P-gp-mediated drug resistant KBv200 cells to paclitaxel and docetaxel by approximately 10-fold *in vitro* and in xenograft models bearing the KBv200 tumors [85]. Moreover, tetrandrine almost completely reversed resistance to vincristine in MDR KBv200 cells *via* direct binding to P-gp to increase intracellular vincristine accumulation in a concentration-dependent manner *in vitro* and *in vivo*, but it had no effect on the sensitivity of drug-sensitive KB cells *in vitro* [86]. Additionally, tetrandrine exhibited an obvious synergistic cytotoxic effect in P-gp mediated MDR Caco-2 and CEM/ADR5000 cancer cells in combination with the common cancer chemotherapeutic agent doxorubicin by reducing P-gp expression in a concentration-dependent manner [87].

In a P-gp expressing human T lymphoblastoid leukemia MOLT-4 MDR cell line (MOLT-4/DNR), tetrandrine reversed MDR and showed even stronger activity for the reversal of drug resistance to daunorubicin (DNR), vinblastine and doxorubicin than the well-known P-gp inhibitor cyclosporin A [88]. P-gp is also a potential mechanism of chemotherapeutic resistance in acute myeloid leukemia, where it is often overexpressed in myeloblasts. When tetrandrine was combined with DNR, etoposide and cytarabine (TET-DEC) in a multicenter clinical trial for 38 patients with poor risk forms of acute myeloid leukemia, TET-DEC was rather well tolerated in these patients [89]. Tetrandrine can consistently reverse DNR resistance in human chronic myeloid leukemia (CML) cell line K562/A02. This chemo-sensitization enhancement was accompanied by elevated cellular DNR accumulation and DNR-induced apoptosis by down-regulating GCS, which has a positive correlation with P-gp [90]. In addition, tetrandrine markedly inhibited the overexpression of doxorubicin-induced mdr1 mRNA/P-gp in K562 cells mediated by inhibiting the doxorubicin-induced expression of NF-kappaB, which is accompanied by the attenuation of NF-kappaB DNA-binding activity [91]. Moreover, tetrandrine citrate has the capacity to inhibit the growth of IM-resistant CML K562, primary leukemia, and primitive CD34 (+) leukemia cells. The antitumor activity against IM-resistant K562 cells and CML cells was induced by the G1 arrest of leukemia cells involved in the depletion of p210 (Bcr-Abl) mRNA and β-catenin protein [92].

The blood-brain barrier (BBB) and MDR are the main causes for poor prognosis of glioma patients after chemotherapy. The lactoferrin-conjugated biodegradable polymersome holding doxorubicin and tetrandrine (Lf-PO-Dox/Tet) antitumor agents loaded into a drug delivery system demonstrated the strongest cytotoxicity against C6 glioma cells and had a greater uptake index with C6 cells than PO-DOX, PO-Dox/Tet, or Lf-PO-Dox by integrating both BBB and glioma-targeting moieties and an MDR inhibitor [93]. For human ovarian cancer and human esophageal squamous carcinoma, MDR is also one of the major causes limiting the efficacy of chemotherapeutic agents. Tetrandrine significantly enhances the cytotoxicity of cisplatin in ovarian cancer, with growth suppression and apoptosis induction by the modulation of the Wnt/cadherin signaling pathway [94]. Tetrandrine adds to the cytotoxic effects of cisplatin in a dose-dependent manner in the human esophageal squamous carcinoma cisplatin-resistant cell line YES-2/DDP, which was isolated by stepwise selection using increasing concentrations of cisplatin. The mechanism underlying this is the down-regulation of MRP1 and the reversal effect on MRP1 activity [95]. Sorafenib is a molecularly targeted agent. It is a potent inhibitor against Raf kinase and several receptor tyrosine kinases, and it has been approved for the clinical treatment of advanced renal and liver cancer [96]. Resistance to sorafenib is a major reason for the failure of anti-hepatocellular carcinoma therapies [97]. Tetrandrine can enhance sorafenib-induced apoptosis in human hepatoma cell lines (BEL7402 and FHCC98), a hepatoblastoma cell line (HepG2), and multiple human colon cancer cells (HCT116, RKO, DLD1) *in vitro* and *in vivo*. Intracellular ROS and Akt activity are involved in the synergistic antitumour activity [52]. For arsenic trioxide, a traditional agent to treat leukemia [98, 99], tetrandrine enhanced the apoptosis, necrosis and cell cycle arrest in As_2_O_3_-treated HepG2 and A549 cells [100]. Tetrandrine also exhibited a synergistic caspase-dependent apoptotic cell death effect in combination with chloroquine, which was widely clinically utilized to treat malaria and other diseases [101], in human hepatoma cell lines (Huh7 and FHCC98), human glioma cell lines (U87 and U251), human lung cancer cell lines (Calu-1 and A549), and human cervical adenocarcinoma HeLa cells *via* ROS production and p21CIP1/WAF1 expression [75]. Endostar was approved for the treatment of cancer as an antiangiogenic agent with limited therapeutic effects when used alone in cancer treatment. However, the combination of tetrandrine and Endostar had a synergistic effect on the antiproliferation of human umbilical vein endothelial cells (HUVECs) and human colon cancer LoVo cells. Furthermore, all these antiangiogenic effects, such as inhibition of cell migration, suppression of tube formation, induction of cell apoptosis, and cell cycle arrest, were enhanced when the HUVECs were treated with tetrandrine combined with Endostar [102]. Tetrandrine also exhibits synergistic anticancer capability with 5-FU to reduce migration and invasion of HCT116 cells [39].

Some derivatives of tetrandrine also displayed anti-MDR activity in cancer cells, and the ability of MDR reversal may be more effective than the drug prototype, tetrandrine. 5-Bromotetrandrine (BrTet), a brominated derivative of tetrandrine, reversed doxorubicin resistance in MDR human breast cancer MCF-7/Dox cells in a dose-dependent manner. BrTet also reversed vincristine, doxorubicin and paclitaxel resistance in MDR KBv200 human oral epidermoid carcinoma cells and innate vincristine and doxorubicin resistance in Bel7402 human hepatocellular carcinoma cells. These activities may be associated with the inhibition of P-gp overexpression and the increase in remaining intracellular anticancer chemotherapeutics [86]. There is a series of new bisbenzylisoquinoline alkaloids partially synthesized from tetrandrine that exhibited an ability to reverse P-gp-mediated MDR in MDR cancer cells (Bel7402 and HCT8) [103]. H1 is a novel derivative of tetrandrine, which displayed favorable anti-MDR activity in KB and KBv200 cancer cells *in vitro* and *in vivo*. Its mechanism may be related to the initiation of the intrinsic apoptosis pathway and the inhibition of Erk1/2 and Akt1/2 activation [26]. PY35 is also a novel 5-substituted tetrandrine derivative. The ability of PY35 to reverse drug resistance was confirmed ina study where PY35 reversed P-gp-mediated resistant K562/Adriamycin (ADM), MCF-7/ADM cells by the co-administration of PY35 and ADM, which increased the intracellular accumulation of drugs, but not by inhibiting the expression of P-gp [104].

## ENHANCING RADIATION SENSITIZATION

Concurrent treatment with chemotherapy and radiation has the potential to offer patients the combined benefits of improved disease control. In basic and clinical research, the anti-tumor agents can be combined with not only other chemotherapeutic agents but also with radiation to sensitize the cells to radiation for enhanced cancer treatment efficacy.

Tetrandrine can increase radiosensitivity by abrogating radiation-induced G2 arrest in the CNE nasopharyngeal carcinoma cell line to increase apoptosis [105]. Moreover, tetrandrine enhanced the lethal effect of radiation on p53-mutant MCF-7/ADR, HT-29 cells and human esophageal cancer TE1 cells in a concentration-dependent manner, which may also involve the relief of radiation-induced G2/M arrest. Additionally, tetrandrine can boost the cell killing activity of irradiation both *in vitro* and *in vivo* [106, 107].

## NANOPARTICLE DELIVERY SYSTEM FOR TETRANDRINE

There have been challenges in conventional chemotherapy, including nonspecific targeting, lack of water solubility, poor oral bioavailability, low therapeutic indices, cancer cell drug resistance, and severe systemic side effects [108, 109]. Nanotechnology is a way to solve this problem and specifically involves the use of bio-conjugated molecular components and engineered materials-nanoparticles for the delivery and targeting of anticancer drugs in cancer cells [110, 111] while avoiding toxicity in normal cells [112, 113]. Accumulated evidence has raised the possibility of developing nanoscale delivery systems using a Trojan horse strategy to achieve improved solubility, stability and cytotoxicity of lipophilic compounds. Because the application of tetrandrine is limited because of its insolubility, it is necessary to explore a tetrandrine delivery system for its use as a cancer chemotherapeutic.

To establish a satisfactory delivery system for the local delivery of tetrandrine, four types of core-shell nanoparticles were prepared from a di-block copolymer of methoxy poly(ethylene glycol)-polycaprolactone (MePEG-PCL) and a tri-block copolymer of polycaprolactone-poly(ethylene glycol)-polycaprolactone (PCL-PEG-PCL). All four types of copolymers exhibited remarkable antitumor effects in LoVo colon cancer cells *in vitro*. Furthermore, the nanoparticles prepared from the di-block copolymer with a particle size of approximately 300 nm and a hydrophobic composition of approximately 80% were the most effective drug carriers for use in further studies [114]. First, a simple way to produce tetrandrine-loaded nanoparticles (Tet-NPs) was to use an amphiphilic block copolymer. In LoVo cells, doses of Tet-NPs at the lower concentrations (1-8 μg/ml) led to more cell inhibition than equivalent doses of free Tet did (1-8 μg/ml). Therefore, further study indicated that the higher the uptake efficiency, the more ROS are generated and the stronger the activation of ROS-dependent c-JNK and caspase-3 induced by the equivalent dose of tetrandrine delivered by nanoparticles [41]. Moreover, cancer microenvironmental factors are important in anticancer drug resistance. Thus, Tet-NPs show superiority as a nanoscale drug carrier as they can withstand pH-induced physiological drug resistance. When the extracellular pH decreased from 7.4 to 6.8, the cytotoxicity of free tetrandrine decreased prominently, but the cytotoxicity of the Tet-NPs was not significantly influenced by pH reduction. *In vivo* experiments also revealed that Tet-NPs reversed PIPDR more effectively than other existing methods and with fewer side effects [115]. Ptx is one of the most widely used anticancer agents and has demonstrated extraordinary activities against a variety of solid tumors. However, the therapeutic response of Ptx is often associated with severe side effects caused by its nonspecific cytotoxic effects. Studies have shown that tetrandrine and Ptx have synergistic antitumor effects against gastric cancer cells. Because the cellular chemo-resistance to Ptx correlates with intracellular antioxidant capacity and tetrandrine possesses the capacity to effectively induce intracellular ROS production, which can deplete Ptx-induced cellular antioxidants, the cytotoxicity of Ptx can be enhanced. In addition, tetrandrine can also increase the stability of Ptx-loaded nanoparticles when tetrandrine is coencapsulated with Ptx into mPEG-PCL nanoparticles. Therefore, nanoparticle codelivery of Ptx and tetrandrine provides a novel therapeutic strategy against gastric cancer [116]. The encapsulation of tetrandrine and Ptx into nanoparticles retains the synergistic anticancer efficiency of tetrandrine and Ptx against mice hepatoma H22 cells as well. When delivered intratumorally, Ptx/Tet nanoparticles exhibited significantly improved antitumor efficacy in the *in vivo* evaluation as intratumoral administration was adopted to increase site-specific delivery. Moreover, the combination substantially increased the overall survival in an established H22-transplanted mouse model [117]. Thus, Ptx/Tet-coloaded nanoparticles could be a potentially useful chemotherapeutic formulation for liver cancer therapy. Furthermore, delivering tetrandrine in PVP-b-PCL nanoparticles *via* endocytosis also leads to enhanced induction of apoptosis in the A549 non-small cell lung cancer cell line; this enhanced apoptosis is achieved by inhibiting the expression of anti-apoptotic proteins. Additionally, Tet-NPs more efficiently inhibit cell migration and invasion than free tetrandrine by down-regulating matrix metalloproteinase (MMP)-2 and MMP-9 as well as up-regulating tissue inhibitor of MMP-3 (TIMP-3) [118]. Therefore, nanodrug delivery systems are an effective way to improve the anticancer efficiency of tetrandrine alone or in combination with other agents.

## EFFECTS OF TETRANDRINE ANALOGS AND OTHER BISBENZYLISOQUINOLINE ALKALOID DERIVATIVES ON CANCER CELLS

The bisbenzylisoquinoline alkaloids are a large family of natural phytochemicals found in medicinal plants that have shown great potential for traditional clinical application in China and other countries. In addition to tetrandrine, cissampareine, a bisbenzylisoquinoline alkaloid from the Cissampelos sp., was identified as cytotoxic for tumor inhibition as early as 1965 [119]. Cepharanthine, the major bisbenzylisoquinoline alkaloid component of *S. epigaea*, exhibited *in vitro* cytotoxicities against multiple human cancer cell lines (A-549, HL-60, MCF-7, SMMC-7721, and SW480) [120]. The antiproliferative activity of cepharanthine alone or in combination with chemotherapeutic vinca alkaloid agents (vincristine, vinblastine, and vindesine) was shown in human colon cancer RPMI 4788 cells and human uterine cervical cancer HeLa cells [121]. Cepharanthine and another alkaloid berbamine completely or partially overcome the resistance of multidrug-resistant human KB carcinoma ChR-24 cells to anticancer agents (vincristine, actinomycin D, daunomycin, and adriamycin) [122]. Another derivative, named neferine, is the major bisbenzylisoquinoline alkaloid derived from the seed embryo of *Nelumbo nucifera* (lotus). Neferine induced G1 cell cycle arrest and apoptosis in A549 cells with the ROS/MAPKs, as well as mitochondrial/caspase cascade activation [123]. In addition, neferine exhibited cytotoxicity against HCC Hep3B cells by inducing apoptosis, autophagy and reducing migration through multiple signaling cascades [124]. Additionally, most studies have revealed the therapeutic potential of fangchinoline, an alkaloid derived from the dry roots of *Stephania tetrandra* S Moore (Menispermaceae). It can inhibit the proliferation of MCF-7 and MDA-MB-231 human breast cancer cells and PC3 human prostate carcinoma cells *via* G1-phase arrest [125, 126] and induce apoptosis in breast cancer cells via the mitochondrial apoptotic pathway and Akt/GSK-3beta/cyclin D1 signaling [127, 128]. Fangchinoline effectively suppressed the proliferation and invasion of lung cancer A549 cells by inhibiting p-FAK and its downstream pathways [129] and induced autophagic cell death *via* p53/sestrin2/AMPK signaling in hepatocellular carcinoma HepG2 and PLC/PRF/5 cells [130]. Notably, fangchinoline has similar effects to tetrandrine as it can reverse the multidrug resistance of cancer cells by inhibiting P-gp activity [87, 131, 132], as well as CBT-1, a bisbenzylisoquinoline plant alkaloid currently in development as a P-gp inhibitor [133]. O-(4-Ethoxyl-butyl)-berbamine, a novel calmodulin antagonist and bisbenzylisoquinoline alkaloid derivative, also improved the chemosensitivity of P-gp-mediated multidrug-resistant cells to doxorubicin by not only blocking the function of P-gp but also inhibiting the expression of P-gp [134]. Taken together, these data suggest that other bisbenzylisoquinoline alkaloid derivatives may have potential applications in cancer therapy, just as tetrandrine does.

## DISCUSSION

Tetrandrine is a calcium channel blocker that inhibits voltage-gated Ca2+ channels [135]. By inhibiting endosomal calcium channels, research has recently shown that tetrandrine inhibits Ebola virus infection both *in vitro* and *in vivo* [18]. Tetrandrine can also inhibit lipid peroxidation, blocking ROS production to protect several types of cells from oxidative stress [136]. Recent examples provide further proof of principle that increasing ROS, whether by increasing production or inhibiting antioxidants, is a promising approach for targeting cancer cells [137]. Inconsistent with the previous ROS blocking function, tetrandrine exhibits anti-tumor effects by trigging ROS accumulation. Because the induction of autophagy can promote the differentiation of cancer cells [138], tetrandrine may be developed to be a differentiation agent, and research in our lab has made marked progress.

Tetrandrine plays important roles in regulating tumor cellular functions, including proliferation/cell cycle, survival/apoptosis, DNA damage repair, cell metabolism, cell motility, and drug resistance. Other bisbenzylisoquinoline alkaloid derivatives such as cepharanthine, neferine and fangchinoline also have multiple therapeutic effects on cancer cells. Therefore, tetrandrine appears to have the potential to be developed as an anti-tumor chemotherapeutic drug. However, this is based on the premise of investigating the directly underlying targets of tetrandrine on cancer cells as the different cancer cells exhibit different cytotoxic effects. To well apply the potential clinical efficacy of tetrandrine for cancer therapy, more mechanism-based pharmacological, metabolism, pharmacokinetic and toxicology studies are required.
